# Early-onset behavioral and neurochemical deficits in the genetic mouse model of phenylketonuria

**DOI:** 10.1371/journal.pone.0183430

**Published:** 2017-08-29

**Authors:** Elena Fiori, Diego Oddi, Rossella Ventura, Marco Colamartino, Alessandro Valzania, Francesca Romana D’Amato, Vibeke Bruinenberg, Eddy van der Zee, Stefano Puglisi-Allegra, Tiziana Pascucci

**Affiliations:** 1 Department of Psychology and Centro “Daniel Bovet”, Sapienza University, Rome, Italy; 2 Cell Biology and Neurobiology Institute, National Research Council, Rome, Italy; 3 European Brain Research Institute EBRI, Rome, Italy; 4 Fondazione Santa Lucia, IRCCS, Rome, Italy; 5 Molecular Neurobiology, GELIFES, University of Groningen, Groningen, The Netherlands; Universita degli Studi Roma Tre, ITALY

## Abstract

Phenylketonuria (PKU) is one of the most common human inborn errors of metabolism, caused by phenylalanine hydroxylase deficiency, leading to high phenylalanine and low tyrosine levels in blood and brain causing profound cognitive disability, if untreated. Since 1960, population is screened for hyperphenylalaninemia shortly after birth and submitted to early treatment in order to prevent the major manifestations of the disease. However, the dietetic regimen (phenylalanine free diet) is difficult to maintain, and despite the recommendation to a strict and lifelong compliance, up to 60% of adolescents partially or totally abandons the treatment. The development and the study of new treatments continue to be sought, taking advantage of preclinical models, the most used of which is the PAH^enu2^ (BTBR ENU2), the genetic murine model of PKU. To date, adult behavioral and neurochemical alterations have been mainly investigated in ENU2 mice, whereas there are no clear indications about the onset of these deficiencies. Here we investigated and report, for the first time, a comprehensive behavioral and neurochemical assay of the developing ENU2 mice. Overall, our findings demonstrate that ENU2 mice are significantly smaller than WT until pnd 24, present a significant delay in the acquisition of tested developmental reflexes, impaired communicative, motor and social skills, and have early reduced biogenic amine levels in several brain areas. Our results extend the understanding of behavioral and cerebral abnormalities in PKU mice, providing instruments to an early preclinical evaluation of the effects of new treatments.

## Introduction

Phenylketonuria (PKU; McKusick2610600) is the most common metabolic inherited disease caused by a deficiency in phenylalanine (Phe) metabolism, resulting in high Phe and low tyrosine levels [1] and in a wide range of symptoms among wich the most common is learning disability [2, 3].

Since 1960, newborn screening and early treatment (a Phe-free diet) have considerably improved outcomes, such that early treated children can lead nearly normal lives. Treatment consists in a low-protein diet supplemented with generally unpleasant Phe-free mixture of amino acids and nutrients that must be consumed in large amount. Although this is still the best therapeutic option, the diet involves major alterations of the lifestyle, and patients and their families experience difficulty in maintaining compliance to dietetic regimen. Moreover, dietary therapy can be associated with deficiencies of several nutrients, some of which may be harmful to brain development, and consolidated evidence indicates that even early-treated PKU patients show cognitive deficits caused by mildly elevated blood Phe levels [4, 5, 6, 7]. Despite the recommendation to stay on dietary treatment for as long as possible, the compliance to the diet gradually decreases after infancy. By adolescence, 60 to 80% of the patients have abandoned the treatment, partially or totally.

The pressure created by these difficulties has renewed the interest in identifying different approaches. During past years, treatment strategies alternative to the diet (e.g. sapropterin dihydrochloride administration, supplementation with large neutral amino acids, gene therapy, treatment with Phenylalanine ammonia lyase) have been proposed and submitted to preclinical studies [8, 9, 10, 11] and clinical trials [12, 13, 14].

The development and the study of new treatments take advantage of preclinical models, the most used of which is the PAH^enu2^ (ENU2), the genetic murine model of PKU. The chemically induced genetic mutation [15] in the chromosome coding for PAH in this model results in a phenotype closely resembling untreated human PKU, being characterized by reduced PAH activity, PHE plasma levels 10–20 times higher than those of healthy littermates, hypomyelination, behavioral and neurochemical deficits [16, 17, 18, 19, 20, 21, 22, 23, 24, 25, 26, 27, 28].

To be efficacious, PKU treatment must start as soon as possible in the early days of life in order to prevent neurodevelopmental perturbations [29].

Previously, we identified the 3^rd^ week of postnatal life as a critical window of brain amine availability in ENU2 mice [22] demonstrating that some behavioral and morphological abnormalities can be rescued by restoration of serotoninergic brain levels between postnatal day (pnd) 14 and 21 [28]. This time window is also critical for synapse formation, dendritic growth and remodelling, axonal refinement and columnarization in rodent cortices [30, 31, 32, 33]. Those data together made it possible to identify a critical period for brain development and thus for therapeutic interventions.

However, to the best of knowledge, no data are available on the behavioral phenotype of ENU2 mice during early postnatal life, as studies focused on adult animals [16, 23, 24, 27, 28, 34].

In order to investigate early behavioral abnormalities in ENU2 mice, we conducted a large set of behavioral phenotyping assay in both males and females of healthy and mutant mice. In particular, we evaluated body measurements, communicative behavior, developmental milestones acquisition, neonatal social recognition, and motor skills development.

Results showed that ENU2 mice are significant smaller than WT, have a different pattern of ultrasonic vocalizations and have a significant delay on the acquisition of several developmental reflexes, never reaching controls’ score in most of them. ENU2 mice also result to have impaired motor and social skills: significant differences were indeed found compared to WT in velocity and distance moved, and in Homing Test ENU2 spend less time in maternal bedding area.

Neurotransmitters level deficits supports behavioral alterations; the biogenic amine levels were found to be reduced in several brain structures in ENU2 pups, with serotonin (5-HT) being the most affected one, as already shown in adult mice.

Our results extend the understanding of brain and behavioral abnormalities in ENU2 mice, providing instruments to evaluate early the effects of new treatments.

## Materials and methods

### Animals

All experiments were conducted per the Italian Law (D. Lgs. 26/14) and the European Communities Council Directive of November 24, 1986 (86/609/EEC) regulating the use of animals for research.

All experiments of this study were approved by the ethics committee of the Italian Ministry of Health and therefore conducted under license/approval ID #: 10/2011-B, according with Italian regulations on the use of animals for research (D. Lgs. 26/14) and NIH guidelines on animal care. Heterozygous multiparous females (+/-) were mated with heterozygous males to obtain -/- ENU2 or +/+ Wild type (BTBR) offspring. Ten days after breeding females were individually housed; delivery day was considered as pnd 0. Nine litters were used, and those made of less than 6 and more than 10 pups were excluded from the experiment. In order to investigate gender-related effects and reduce variability, as well as based on our previous experience maximum 10 animals for sex (M, F) were used for all experiments. All pups were equally manipulated but only the score obtained by two male and female pups per litter were randomly chosen to be included in the statistics, in order to be sure that all the litters were equally represented. The experimenters were unaware of genotypes until pnd 10–12, when ENU2 mice become recognizable from the fur.

Before the beginning of the tests (pnd 3) mice were marked through distal phalanx removal, in order to be recognizable during the whole testing period. For genotyping, tail biopsy was performed; DNA was prepared using the Easy DNA Kit (Invitrogen, Carlsbad, CA, USA), and the PAH mutation on exon 7 was detected by PCR amplification and digestion thought restriction enzyme BsmAI (NewEnglandBiolabs, Inc., U.S.).

Behavioral assessment begun on pnd 4 and finished on pnd 18; animals were then weaned on pnd 28. When possible every tested pup underwent the complete test battery, in order to be able to follow its development.

On pnd 14 six behaviorally naïve male and female pups per strain were sacrificed through decapitation and the brains collected, immediately frozen into liquid nitrogen and stored at -80° until the biochemical analysis.

During mating and breeding mice were provided with food and water ad libitum and were on a 12:12-h dark:light cycle (lights on from 7 AM to 7 PM) and everything was made to minimize animals discomfort.

### Body growth

**Fig 1:**
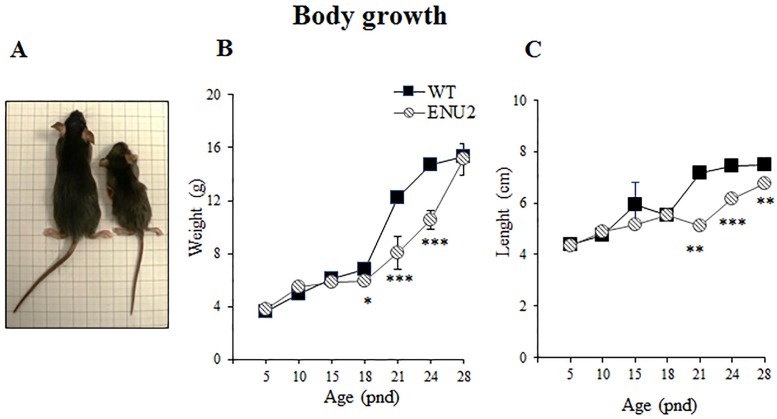
Body growth. Representative photograph (a) of WT (left) and ENU2 (right) mice showing body size differences at pnd 24. Analysis of weight (b) and length (c) of WT and ENU2 from pnd 5 to weaning at pnd 28. Significant differences in body weight were found on pnd 18, 21, 24 and 28, and in length on pnd 21, 24 and 28, ENU2 being smaller than controls. Data are expressed as mean ± SEM. *p < .05, **p < .01, ***p < .001 WT vs ENU2.

Body weights and lengths of pups were measured to investigate offspring growth on pnd 5, 8, 11, 14, 17, 21, 24 and 28 (Fig 1). Data were analyzed by two-way ANOVA for repeated measures (genotype, 2 levels: WT and ENU2 as the between-group factor; age, 8 levels: 5, 8, 11, 14, 17, 21, 24, and 28 as within-group factor; WT: 8 female and 9 male; ENU2: 7 female and 7 male) followed by post-hoc Duncan’s test for multiple comparisons.

### Ultrasound emissions evaluations (USVs)

The ultrasound emission is the main communicative behavior pups display to elicit maternal care. Abnormalities in the typical emission pattern might underlie damaged mother-pup bound, anxiety problems and development impairment. From pnd 4 to 14 to evaluate ultrasounds, after an hour acclimatization in the testing room, pups were firstly separated from the mother and after 5 min individually placed in a clean beaker covered with clean sawdust and the emissions recorded for five minutes. To prevent cooling, pups were maintained on a hot plate (36°) and after recording they were returned to the home cage. No more than 5–6 pups per litter were tested [35].

**Fig 2:**
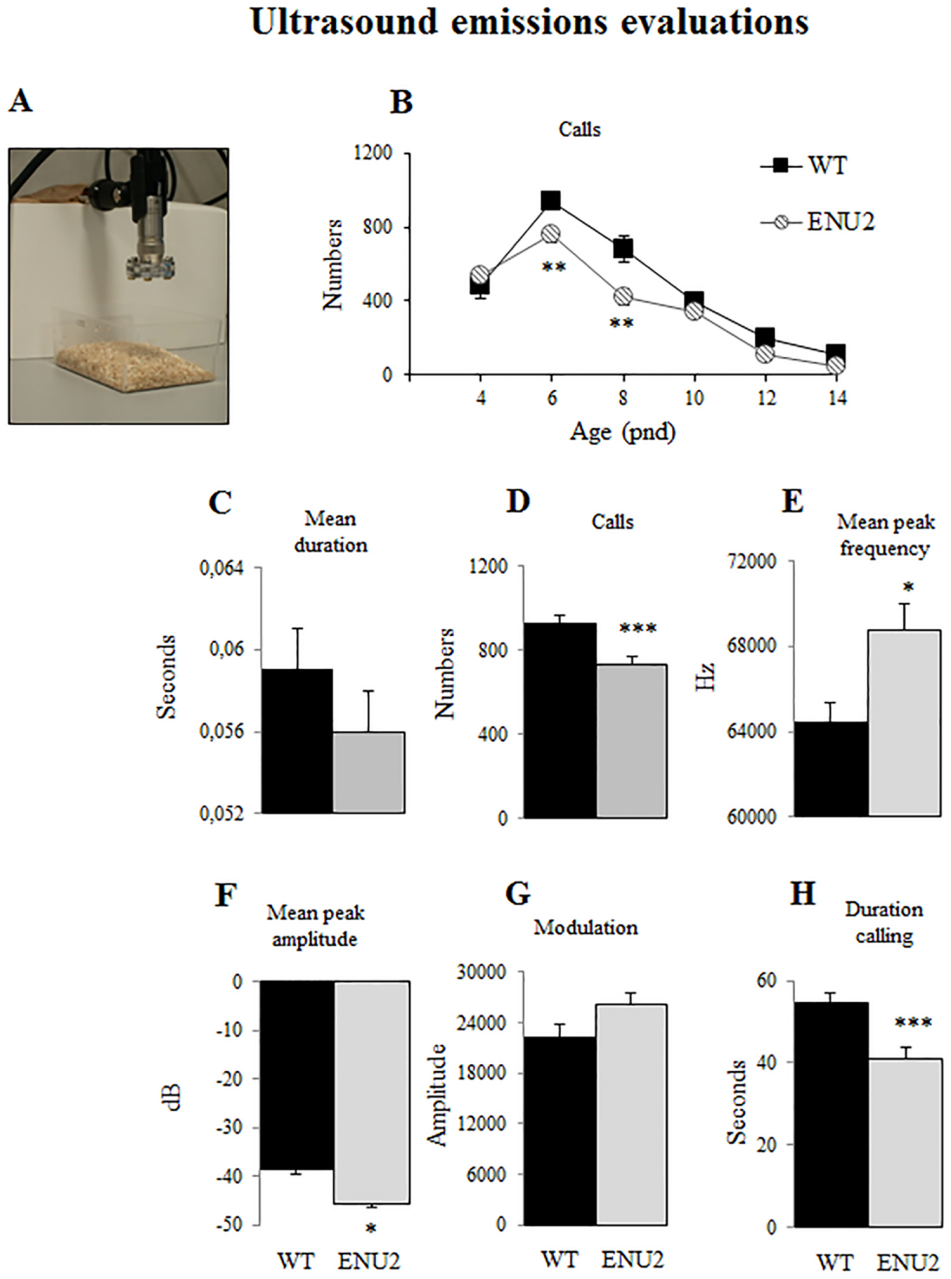
Ultrasound emission analysis. Apparatus (a) and number of vocalization on pnd 4, 6, 8, 12 and 14 (b). Significant strain differences were found on pnd 6 and 8; ENU2 pups never reach the peak present in WT. Analysis of duration (c), number (d), mean peak frequency (e), mean peak amplitude (f), modulation of calls (g) and time spent calling (h) at pnd 6 in response to maternal separation in WT and ENU2 pups. ENU2 emit less calls ad spend less time calling in total, but the emissions are higher in frequency and amplitude. Data are expressed as mean ± SEM. *p < .05, **p < .01, ***p < .001 WT vs ENU2.

The registration apparatus, shown in Fig 2A, consisted of an UltraSoundGate Condenser Microphone (CM16, Avisoft Bioacoustics, Berlin, Germany), sensitive to frequencies of 15–180 kHz, with a flat frequency response (± 6 dB) between 25–140 kHz, was placed 1 cm above the beaker and connected to a computer that recorded data as 250,000 Hz in 16-bit format wav files. Sound files were transferred to SASLab Pro (version 5.2; AvisoftBioacoutics) for sonographic analysis, and fast Fourier transformation was conducted (512 FFT-length, 100% frame, Hamming window, and 75% time window overlap). Spectrograms were produced at 488 Hz frequency resolution and 0.512 ms time resolution. To detect USVs, an automatic threshold-based algorithm and a hold time mechanism (hold time: 20 ms) were used. Signals below 40 kHz were truncated to reduce background noise to 0 dB [36]. Inaccurate detections were adjusted manually by an experienced user [37]. We analyzed the curve of the number of calls during development and on pnd 6 we focused on different features of the calls, such as mean duration, number, mean peak frequency, mean peak amplitude, modulation and total time spent calling.

The curve of the number of USVs emitted was analyzed by two-way ANOVA for repeated measures (genotype, 2 levels: WT and ENU2 as between-group factor; age, 4 levels: 6, 8, 10, 12 as within-group factor; (WT: females and 6 males; ENU2: 5 females and 5 males) followed by post-hoc Duncan’s test for multiple comparisons. The analysis of the USVs features on pnd 6 was performed by one-way ANOVA to evaluate the genotype effect (WT: 8 females and 8 males; ENU2: 7 females and 7 males).

### Developmental reflex acquisition

From pnd 5 to pnd 17 sensorimotor reflexes such as righting reflex, negative geotaxis, screen test, vertical screen test, auditory startle, cliff avoidance, vibrissae stimulation reflex, visual placing response, were recorded [38, 39].

The parameters analyzed were the latency to act out the behavior (righting reflex and negative geotaxis) or the score obtained on a four level scale (0 = no response; 1 = weak response; 2 = incomplete response; 3 = complete/adult-like response)

If no response was present within 30 seconds, the animal was given 0 points, or 30” (cut off time) in the latency evaluation.

Development of reflexes was analyzed by two-way ANOVA for repeated measures (genotype, 2 levels: WT and ENU2 as between-group factor; age, 5 levels: 5, 8, 11, 14, 17 for each reflex, as within-group factor (WT: 12 female and 10 male WT; ENU2: 11 female and 12 male) followed by post-hoc Duncan’s test for multiple comparisons.

### Homing Test

Pups were tested on pnd 10. After an hour habituation in the testing room, mothers were removed from the home cages and placed on a hot plate (36°) to prevent offspring cooling. The test started after 5 min separation.

The testing apparatus was made of a central compartment and 2 lateral arms (31 cm x 4 cm with walls 6 cm in height), one covered with home cage bedding, the other with clean sawdust. Pups were individually placed in the empty center and, after 1 minute of habituation, they had to choose between the two arms. Time spent into the arm containing the nesting litter, percentage of maternal arm choices, latency to reach maternal and zone crossings were scored [35, 39, 40].

A video-based EthoVision system (Noldus, The Netherlands) was used to record and analyze the data. Statistical analysis was performed by one-way ANOVA to evaluate the effect of the genotype on the parameters (WT: 8 female and 9 male; ENU2: 7 female and 7 male).

### Open Field

Animals underwent the Open Field test on pnd 18 to evaluate motor skills development.

Pups were individually tested for five minutes [39, 41, 42]. Distance moved (cm) and velocity (cm/sec) were used as dependent variables. To record and analyze the data we used the EthoVision system (Noldus, The Netherlands) was used.

Statistical analysis was performed by one-way ANOVA (WT: 8 female and 9 male; ENU2: 7 female and 8 male) to evaluate the effect of genotype on the parameters.

### Neurotransmitters analysis

Punches were obtained as previously described [25]. Frozen brains were sliced (coronal sections) and punches of the mpFC, CP, Amy, NAc, and Hip were made with stainless steel tubes of 0.8, 1.0, or 1.5 mm inside diameter.

The punches were stored in liquid nitrogen until the day of the analysis.

Frozen samples were weighed and homogenized in 0.05 M HClO4. The homogenates were centrifuged at 14000 rpm for 20 min at 4°C. Tissue levels of 5-HT, DA, NE and their metabolites 5-HIAA, DOPAC, HVA and MHPG were assessed using HPLC system [25]. The HPLC system consists of an Alliance (Waters) system and a coulometric detector (ESA Model 5200A Coulochem II) provided with a 5011 high sensitivity analytical cell and a 5021 conditioning cell, the potential being set at .450mV and .100 mV, respectively. The column, a Nova-Pack Phenyl column and a Sentry Guard Nova-Pack pre-column, were purchased from Waters Assoc. The flow rate was 1 ml/min. The mobile Phase consisted of 3% methanol in 0.1M Na-phosphate buffer pH 3, 0.1mM, Na2 EDTA and 0.5mM 1-octane sulphonic acid Na salt. Statistical analysis of for the effect of genotype on the single structure and compound was performed by one-way ANOVA.

## Results

Since no significant difference between male and female was found in all the tests, the results are shown as sex-balanced groups.

### Reduced body growth

Body growth was measured from pnd 5 to 28 (Fig 1A, 1B and 1C). Statistical analysis revealed significant genotype x age interaction for weight (F_(6,174)_ = 8,149; p<0.001; post hoc analysis p < .05 and .001 for ENU2 to WT at pnd 18, 21 and 24; Fig 1B) and length (F_(6,174)_ = 28,165; p<0.001; post hoc analysis p < .001 and .05 for ENU2 to WT at pnd 21, 24 and 28; Fig 1C).

ENU2 pups are shorter and weight less than WT, and these differences become significant at the beginning of the weaning, when maternal nourishing is reduced.

### Reduced ultrasound emission

Measurements of ultrasonic emissions in separated pups (curve of emissions from pnd 4 to pnd 14 Fig 2B; pnd 6, Fig 2C, 2D, 2E, 2F, 2G and 2H) showed significant differences between WT and ENU2 mice. Two-way ANOVA revealed significant genotype x age interaction for the number of calls (F_(5,100)_ = 2,898; p<0,05; post hoc analysis p < .001 for ENU2 to WT at pnd 6 and 8): ENU2 pups emit significant fewer calls and never reach the peak present in WT calls, although a curve of emissions is still present.

One-way ANOVA revealed that at pnd 6 ENU2 mice emit less calls in comparison with WT (F_(1,28)_ = 14,788; p<0,001), the calls emitted have a higher mean peak frequency (F_(1,28)_ = 7,971; p<0,01) and a lower mean peak amplitude (F_(1,28)_ = 11,063; p<0,01), and, even if the difference in the mean duration of the calls is not significant, the total amount of time spent calling is significantly lower (F_(1,28)_ = 13,539; p<0,001).

### Delay in the developmental reflex acquisition

**Fig 3:**
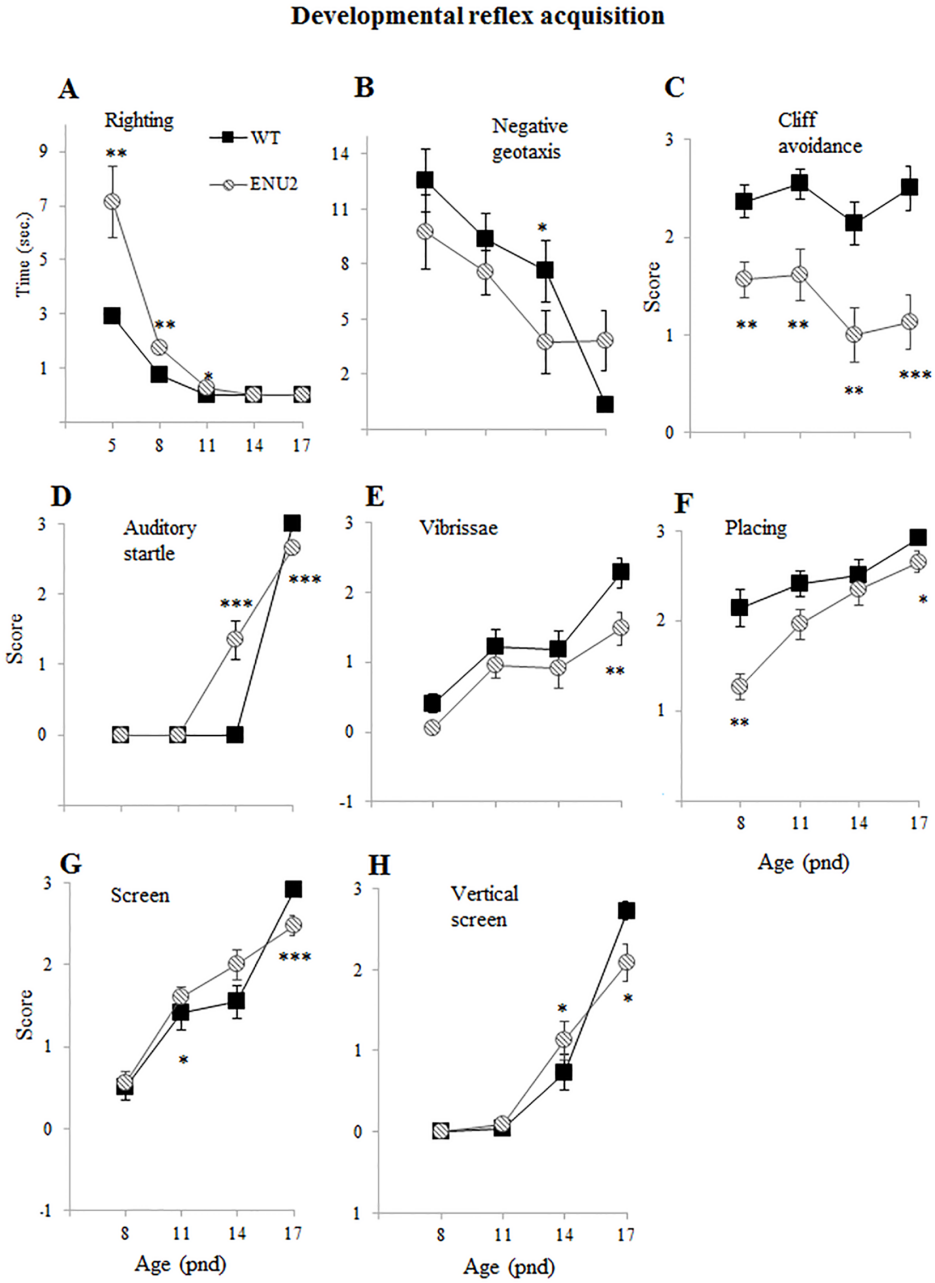
Developmental reflex assay. Analysis of acquisition of the somatosensorial reflex righting (a), negative geotaxis (b), cliff avoidance (c) auditory startle (d), vibrissae stimulation reflex (e), visual placing response (f), screen test (g), vertical screen test (h) in WT and ENU2 mice on pnd 5, 8, 11, 14 and 17. ENU2 show delayed acquisition of all tested reflexes, indicating impaired motor and sensory skills. Data are expressed as mean ± SEM. *p < .05, **p < .01, ***p < .001 WT vs ENU2.

Fig 3 shows the acquisition of early (from pnd 5 to 17) developmental reflexes in ENU2.

#### Righting reflex

(Fig 3A), that is the latency to turn over to the upright position when placed on the back, was tested at pnd 5 to 17 in both groups. Statistical analysis revealed a significant genotype x age interaction on righting reflex latency (F_(4,168)_ = 7,783; p<0.001; post hoc analysis p < .001 and .05 for ENU2 to WT at 5, 8 and pnd 11).

#### Negative geotaxis

(Fig 3B), that is the latency to change the orientation when placed on an inclined plan, was tested at pnd 8 to 17 in ENU2 ant WT mice. Statistical analysis revealed a significant genotype x age interaction (F_(3,126)_ = 2,676; p<0.05; post hoc analysis p < .05 for ENU2 to WT at pnd 14)

#### Cliff avoidance

(Fig 3C), the ability of the pup to moving away when placed on the edge of a raised platform, was recorded from pnd 8 to 17 and statistical analysis revealed significant genotype effect (F_(1,126)_ = 45,504; p < .001) but no significant interaction with age.

#### Auditory startle

(Fig 3D), i.e. the response to acoustic stimuli, was recorded from pnd 8 to pnd 17, and statistical analysis showed a significant genotype x age interaction (F(3,126) = 24,771; p<0.001; post hoc analysis p < .001 for ENU2 to WT at pnd 14 and 17).

#### Vibrissa placing response

(Fig 3E), measured from pnd 8 to 17 by evaluating the response of pup to raise head and forelimbs to grasp a cotton swab after vibrissae stimulation, revealed only a significant genotype (F_(1,126)_ = 5,777; p < .05) and age effect (F_(3,126)_ = 23,177; p < .001).

#### Placing reflex

(Fig 3F), i.e. ability of the pup to rise his head and extend the forelimbs toward a solid surface after being suspended by the tail, has been measured from pnd 8 to 17, and statistical analysis revealed significant genotype x age interaction (F_(3,126)_ = 20,330; p<0.001; post hoc analysis p < .01 and .05 at pnd 8 and 17).

#### Level screen test

(Fig 3G) has been analyzed by measuring from pnd 8 to 17 if the pup can hold onto a screen when it is horizontally dragged by the tail across it, and statistical analysis showed significant genotype x age interaction (F_(3,126)_ = 65,192; p<0.001; post hoc analysis p < .05 and .001 pnd 11 and 17).

#### Vertical screen test

(Fig 3H) measures the climbing response of the pup when the wire mesh screen in which is placed is rotated in a vertical position; the ANOVA revealed significant effect of genotype x age interaction (F_(3,126)_ = 6,042; p<0.001; post hoc analysis p < .05 and .001 at pnd 14 and 17).

Altogether, analyzed data showed that ENU2 mice have impairments in almost all tested skills, showing a motor and sensory developmental delay.

### Alterations in the Homing Test

**Fig 4:**
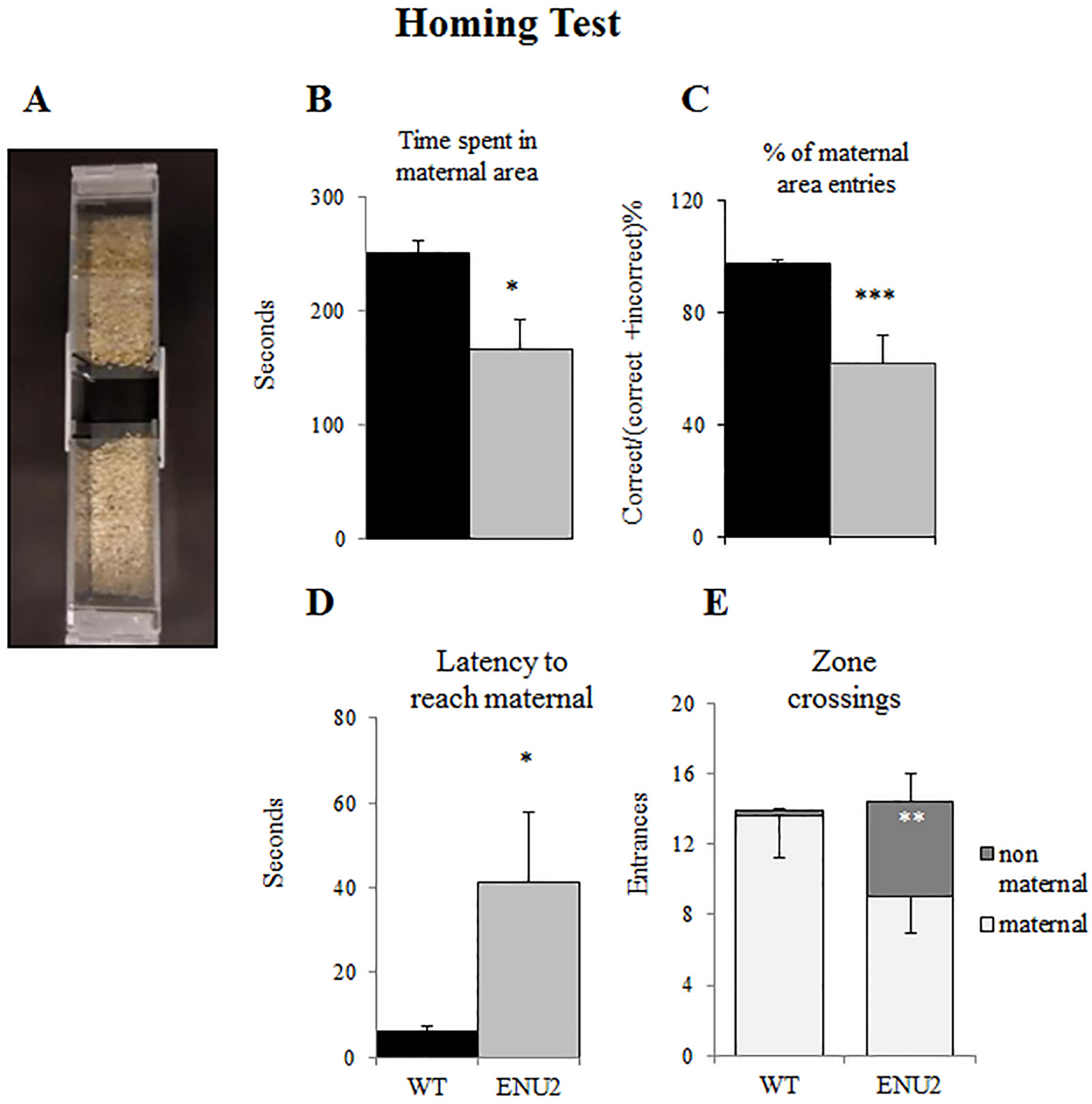
Homing Test. Homing test apparatus (a) and analysis of differences in time spent in maternal area (b), % of maternal area entries (c), latency to reach maternal (d) and zone crossing (e) in WT and ENU2 pups on PND 10.ENU2 performances are significantly worse than WT, indicating slower development of social cognitive abilities. Data are expressed as mean ± SEM. *p < .05, **p < .01, ***p < .001 WT vs ENU2.

A significant genotype effect was found on the percentage of the choice of the area containing maternal litter sawdust (Fig 4A, 4B, 4C, 4D and 4E). ANOVA demonstrated that ENU2 10-day-old pups spent less time in the nest area in comparison with WT (F_(1,29)_ = 14,135; p<0,05); also the percentage of entries in the nest area was significantly different (F(1,30) = 5,309; p < .001). Significant genotype effect was also found in latency of entries in the maternal area (F_(1,35)_ = 6,516; p < .05), likewise significant was the difference in the entries in the non-maternal area (F_(1,29)_ = 13,147; p < .01) shown after the analysis of the apparatus zone crossing.

### Deficits in the Open Field Test

**Fig 5:**
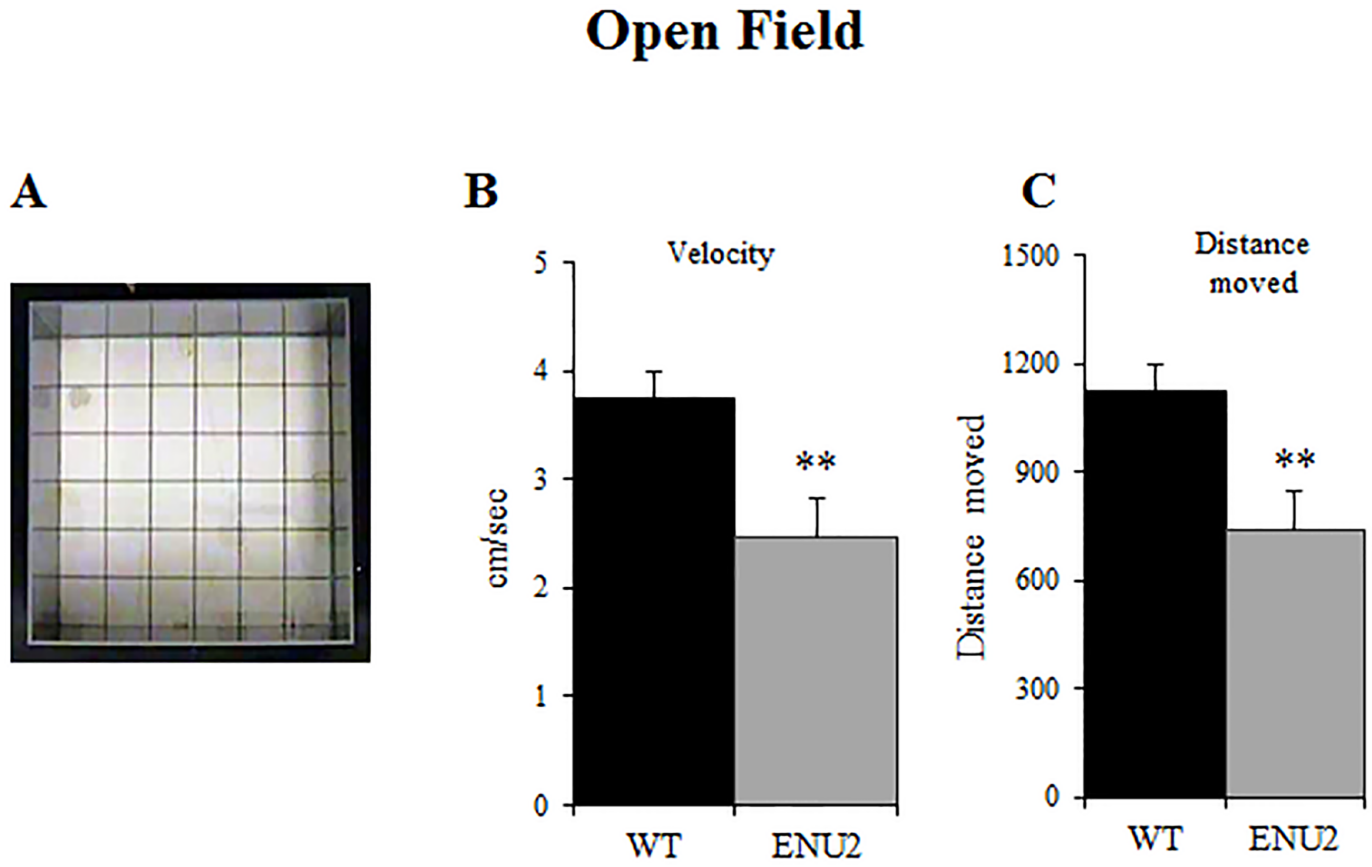
Open Field. Open Field apparatus (a) and analysis of the differences between WT and ENU2 in velocity (b) and distance moved (c). ENU2 were found to be slower and moved less than WT. Data are expressed as mean ± SEM. **p < .01 WT vs ENU2.

Locomotor activity of 18-day-old pups in an empty Open Field was lower in ENU2 mice compared to WT (Fig 5A, 5B and 5C). In particular, statistical analysis revealed significant genotype effect on velocity (F_(1,30)_ = 9,250; p<0,01) and distance moved (F_(1,30)_ = 9,213; p<0,01) parameters, showing that at pnd 18 ENU2 are significantly slower and move less than healthy control mice.

Center time in the apparatus did not differ between groups.

### Early neurochemical abnormalities

Results from analysis of neurotransmission in different brain areas (medial prefrontal Cortex, *mpFC*; Nucleus Accumbens, *NAC*; Caudate Putamen, *CP*; Hippocampus, *HIPP*; Amygdala, *AMY*) are reported in Table 1.

**Table 1:** Biogenic amines and metabolites tissue levels (ng/g wet weight) in different brain areas of BTBR and ENU2 mice at PND 14.

		Area				
		mpFC	CP	Amy	Hipp	NAc
**5-HT**	WT	36 ± 8	532 ± 111	122 ± 36	50 ± 6	31 ± 8
	ENU2	**10 ± 2** ***	241 ± 71*	26 ± 7**	17 ± 4***	5 ± 2**
**5-HIAA**	WT	78 ± 10	568 ± 115	1662 ± 458	429 ± 110	300 ± 72
	ENU2	13 ± 2***	61 ± 9***	374 ± 50**	148 ± 26***	29 ± 6**
**NE**	WT	118 ± 11	71 ± 8	189 ± 39	306 ± 43	1282 ± 479
	ENU2	81 ± 15	37 ± 8**	91 ± 18*	185 ± 42	316 ± 78
**MHPG**	WT	5 ± 1	4 ± 1	32 ± 7	-	209 ± 237
	ENU2	4 ± 1	1 ± 0, 4*	18 ± 2	-	97 ± 50
**DA**	WT	112 ± 11	1836 ± 476	264 ± 80	108 ± 46	2879 ± 990
	ENU2	20 ± 3*	1357 ± 191	79 ± 21*	111 ± 30	614 ± 185**
**DOPAC**	WT	7 ± 1	364 ± 63	97 ± 35	56 ± 9	330 ± 48
	ENU2	6 ± 1	354 ± 96	45 ± 9	52 ± 13	160 ± 43*
**HVA**	WT	33 ± 4	272 ± 53	196 ± 59	162 ± 33	728 ± 177
	ENU2	26 ± 4	296 ± 63	92 ± 18	114 ± 23	206 ± 40*

Almost all amine and metabolites result to be deficient in ENU2 in investigated areas, except NE in mpFC, Hipp and NAC and of DA in CP and HIPP; 5-HT and 5-HIAA are the most affected ones in all areas.

Values are expressed as mean± ES.

* p<0,05,

** p<0,01,

*** p<0,001 BTBR vs ENU2.

Data are indicated as mean concentrations of biogenic amines (dopamine, *DA*; norepinephrine, *NE*; 5-hydroxytryptamine, *5-HT*) and metabolites (3-4-Dihydroxyphenylacetic acid, *DOPAC*; homovanillic acid, *HVA*; 3-methoxy-4 hydroxyphenylethyleneglycol, *MHPG*; 5-hydroxyindoleacetic acid, *5-HIAA)* ±ES.

One-way ANOVAs revealed a significant genotype effect for almost all amines in all investigated brain areas, with the exception of NE in mpFC, Hipp and NAC and of DA in CP and HIPP.

As previously evidenced (25, 22, 23, 24), the levels of 5-HT and of its metabolite 5-HIAA were early and significantly reduced in ENU2 mice regardless of the brain areas.

## Discussion

In a murine model of a metabolic inherited disease it is essential to analyze behavioral and neurochemical phenotyping during the early developmental period in order to identify the exact onset of behavioral symptoms and brain alterations, and then to indicate the best time for early intervention and what parameters to keep an eye on during preclinical evaluation of experimental treatments. To date, although behavioral and neurochemical alterations have been extensively demonstrated in adult mice [34, 43], this study is the first to report the onset of these deficiencies, providing a comprehensive behavioral and neurochemical assay of the developing ENU2 mouse, the best murine model of PKU. Our study shows that ENU2 mice reach many developmental milestones later than their healthy littermates. First of all, in mutant mice body weight and length were reduced during the third postnatal week. This result is in line with several reports suggesting that growth in early childhood in PKU is reduced in comparison with reference populations [44], as well as with a previous preclinical report showing reduced postnatal growth in ENU2 mice in comparison with heterozygous littermates [45]. It should be noted that, although heterozygous ENU2 mice have been often used as control group instead of WT subjects, abolishing the costs of genetic characterization following heterozygous mating, recent data have confirmed hyperphenylalaninemic status of heterozygous ENU2 mice [24]. Therefore, the use of heterozygous mice as control group is questioned.

Mice emit and perceive Ultrasound Vocalizations (USVs) to communicate; in particular USVs are emitted by pups when taken out from the nest and separated from their mother, to induce maternal retrieval in the nest. Therefore, USVs are commonly used in preclinical studies to measure communication deficits in murine models of neurodevelopmental disorders. We registered USVs from pnd 4 until pnd 14 to study the whole curve of emissions, and we found that ENU2 pups never reach the peak present in WT calls. Since pnd 6 represents the peak of emissions we decided to go deeper into the characterization. The analysis of different parameters of the emissions at pnd 6 revealed that ENU2 pups emit less call, and even if the mean duration of the calls did not differ between strains, the overall time spent calling is reduced in ENU2; the calls are also “softer”, as indicate by the Amplitude and are more frequent indicating also qualitative differences in the emissions. These data add another aspect to the behavioral profile of EN2 mice, being communication damaged in both quantitative and qualitative features.

Moreover, in every developmental milestones ENU2 mice showed a difference in comparison to WT in terms of reduced performance, in at least one time point. Generally, ENU2 reached fully developed reflexes later than healthy littermates, as showed by increased latency or reduced scores obtained in many of them.

Homing Test allows investigating complex abilities, such as sensory and motor skills, odors recognition and preference for social inputs. In the Homing Test, 10-day-old ENU2 pups showed longer latency to reach, less time spent in and lower percentage of entries in the area containing nesting odor, in comparison to WT. Although ENU2 pups present reduced locomotor activity, in the Homing Test they carry out a number of zone crossings comparable to healthy littermates. Therefore, data suggest slower development of social olfactory and related cognitive abilities as compared to control mice.

In addition, distance moved and velocity during the Open Field Test showed that 18-day-old ENU2 pups had lower general activity than age-matched WT. These data are in agreement with several previous studies showing reduced locomotor activity in adult animal model of PKU [24, 34, 35, 43] and underline the precociousness of the deficit.

According to behavioral data, neurochemical analysis revealed early abnormalities in 14-day-old ENU2 pups. ENU2mouse brain is early characterized by relevant cerebral deficits, with particularly dramatic deficiency in serotonergic metabolism, characterized by more than 50% of level reduction in all investigated brain areas. With respect to catecholamine reduction, the presence and the seriousness of effects depend on the observed cerebral structure.

The brain amine reduction observed here is consistent with clinical and preclinical neurochemical studies showing reduced aminergic metabolism in brain [46] and cerebrospinal fluid [47, 48, 49] of PKU patients and in PKU mouse brain [43]. About prominent 5-HT deficits, this is consistent with previous studies on amine synthesis in hyperphenylalaninemic organisms, in adulthood [24, 25, 50, 51] as well as during early postnatal development [22, 28] in ENU2 mice. Synthesis of 5-HT occurs via tryptophan hydroxylation to 5-hydroxy-tryptophan by tryptophan hydroxylase enzyme. Reduced cerebral 5-HT synthesis may be the result of reduced tryptophan brain concentrations caused by reduced blood-brain-barrier transport of tryptophan or by reduced tryptophan hydroxylase activity at elevated, plasma or brain respectively, Phe concentrations [3]. As a whole, the extent of early neurochemical effects can account for several and early behavioral alterations observed in developing ENU2 mice.

Present study identifies early behavioral and neurochemical alterations in the best murine model of PKU, providing a comprehensive assay of the several deficits in young ENU2 mice, as well as the parameters to keep an eye on during preclinical evaluation of alternative treatments. Our data showing early behavioral and neurochemical characterization of ENU2 strengthens the face validity of the genetic murine model of PKU. Furthermore, these early neurobehavioral alterations make possible, in the future, to evaluate precociously the effects of alternative treatments, monitoring early stages of postnatal life in live hyperphenylalaninemic mice.

## Abbreviations

5-HIAA: acid 5-hydroxyindoleacetic
5-HT: 5-hydroxytryptamine, serotonin
AMY: Amygdala
CP: Caudate Putamen
DA: dopamine
DOPAC: 3-4-Dihydroxyphenylacetic acid
HIPP: Hippocampus
HVA: homovanillic acid
MHPG: 3-methoxy-4 hydroxyphenylethyleneglycol
mpFC: medial prefrontal Cortex
NAC: Nucleus Accumbens
NE: norepinephrine
PAH: phenylalanine hydroxylase
PKU: Phenylketonuria
pnd: postnatal day
USVs: Ultrasound Vocalizations
